# Generation of medaka gene knockout models by target-selected mutagenesis

**DOI:** 10.1186/gb-2006-7-12-r116

**Published:** 2006-12-08

**Authors:** Yoshihito Taniguchi, Shunichi Takeda, Makoto Furutani-Seiki, Yasuhiro Kamei, Takeshi Todo, Takao Sasado, Tomonori Deguchi, Hisato Kondoh, Josine Mudde, Mitsuyoshi Yamazoe, Masayuki Hidaka, Hiroshi Mitani, Atsushi Toyoda, Yoshiyuki Sakaki, Ronald HA Plasterk, Edwin Cuppen

**Affiliations:** 1 Department of Radiation Genetics, CREST, Japan Science and Technology Laboratory, Kyoto University, Yoshida Konoe, Sakyo-ku, Kyoto 606-8501, Japan; 2 Kondoh Differentiation Signaling Project, Exploratory Research for Advanced Technology (ERATO), Japan Science and Technology Corporation, Yoshida-kawaramachi, Sakyo-ku, Kyoto, 606-8305, Japan; 3 Department of Mutagenesis, Radiation Biology Center, Kyoto University, Yoshida Konoe, Sakyoku, Kyoto 606-8501, Japan; 4 Hubrecht Laboratory, Uppsalalaan, Utrecht, The Netherlands; 5 Department of Integrated Biosciences, The University of Tokyo, 5-1-5 Kashiwa-no-ha, Kashiwa, Chiba 277-8562, Japan; 6 The Institute of Physical and Chemical Research Genomic Sciences Center, RIKEN Yokohama Institute, 1-7-22 Suehiro, Tsurumi-ku, Yokohama, Kanagawa 230-0045, Japan

## Abstract

We have established a reverse genetics approach for the routine generation of medaka (*Oryzias latipes*) gene knockouts. A cryopreserved library of *N*-ethyl-*N*-nitrosourea (ENU) mutagenized fish was screened by high-throughput resequencing for induced point mutations. Nonsense and splice site mutations were retrieved for the *Blm*, *Sirt1*, *Parkin *and *p53 *genes and functional characterization of *p53 *mutants indicated a complete knockout of p53 function. The current cryopreserved resource is expected to contain knockouts for most medaka genes.

## Abstract 2

A reverse genetics approach for the routine generation of medaka (*Oryzias latipes*) gene knockouts is described and applied to create a cryopreserved resource containing knockouts for most medaka genes.

## Background

Small laboratory fish such as zebrafish and medaka, the Japanese killifish, are attractive vertebrate animal models that are easy to handle and are ideally suited for genetic studies because of their large numbers of progeny per generation [1]. Furthermore, fish models are being embraced because of their extended similarity in mutagenesis and carcinogenesis processes with rodent models and possibly humans [2]. The development of fish mutants will provide additional tools to explore the mechanisms of these processes.

In forward genetics, the mutated gene that underlies a certain phenotype is identified, while in reverse genetics, the phenotype that results from mutating a given gene is determined. To date, the majority of large-scale genetic studies have been confined to forward genetics [3-5]. Although these studies are very powerful and have been very successful, only conspicuous gene functions can be detected within the limits of the very labor-intensive phenotype-driven assays. Furthermore, biological pathways are often characterized by two or more parallel pathways that support a single biological process (genetic redundancy; reviewed by Tautz [6]). In particular, teleosts underwent a lineage-specific partial or whole genome duplication [7], making it possible that phenotypic consequences of the inactivation of a single gene, as is the case in forward genetic screens, are masked by the action of a paralogous gene(s) with (partial) overlapping functions. Reverse genetics or knockout approaches are well-suited not only to address these issues via the generation of double mutants but also for assigning biological function to uncharacterized genes in a genome. Draft genome sequences for both zebrafish and medaka are already available and many genes with unknown function have been annotated [8].

Morpholino-modified oligonucleotides can be used to inactivate genes in both zebrafish and medaka [9], but there are also some important drawbacks to this approach: first, the knockout effect is transient and diminishes a few days after the injection; second, therefore, there is only very limited application to adult phenotypes; third, morpholinos must be injected into eggs in each individual experiment, over and over again; and fourth, extensive amounts of controls have to be included in every experiment to control for specificity. Permanent gene inactivation by genetic modification would overcome these issues. Although conventional gene targeting in zebrafish embryonic stem (ES) cells using homologous recombination has recently been established *in vitro *[10], no transgenic knockout fish have been generated yet using this approach. Instead, all existing zebrafish knockouts have been generated using a more general target-selected mutagenesis approach [11,12]. The germline of male founder fish was randomly mutagenized using the supermutagen ENU (*N*-ethyl-*N*-nitrosourea) and induced mutations were retrieved from a large library of F1 progeny using PCR-based amplification of target genes of interest, followed by mutation discovery by dideoxy resequencing.

Here, we report the establishment of an efficient target-selected gene inactivation approach for medaka, and demonstrate that the mutations that were retrieved in the *p53 *gene result in a complete loss-of-function phenotype.

## Results and discussion

### Medaka mutant library generation and screening

**Figure 1:**
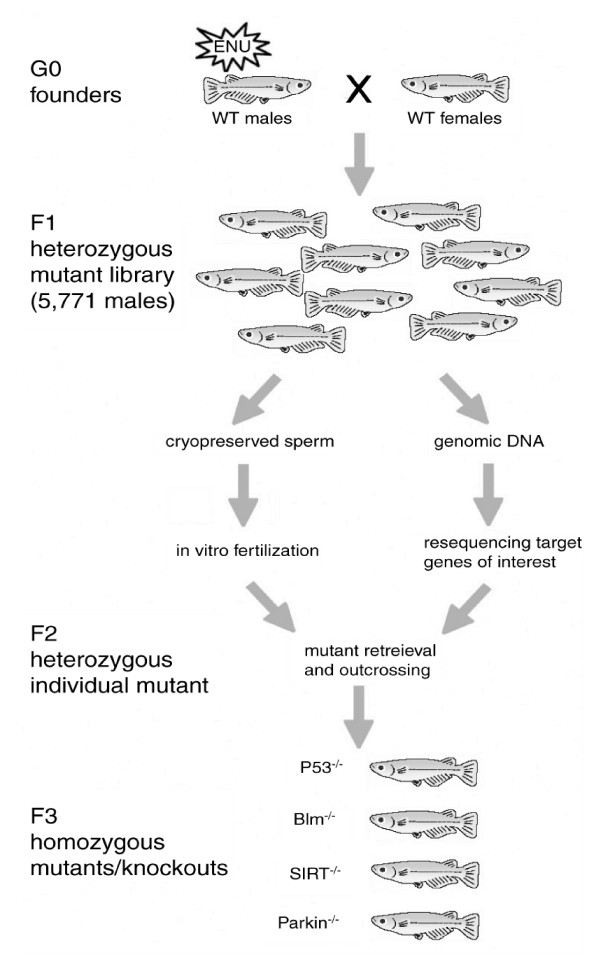
Schematic outline of the mutant medaka library generation and screening. Male G0 fish were ENU-mutagenized and crossed with wild-type (WT) females. Male F1 progeny were used for sperm cryopreservation and parallel DNA isolation. The library was screened for induced mutations in target genes of interest by dideoxy resequencing. Interesting mutants were retrieved from the cryopreserved archive by *in vitro *fertilization and incrossed to homozygosity for phenotypic analysis.

The mutant medaka library was generated and screened as schematically outlined in Figure 1. Founder fish were repeatedly mutagenized with ENU, crossed with wild-type females, and the progeny were used to establish a permanent cryopreserved resource of 5,771 F1 males (Table 1). To get an indication about the induced mutation frequency, we performed a specific locus test using the albino mutant [4]. The appearance of a white-eyed embryo at a rate of 1 in 272 (Table 1) is in line with previously observed frequencies [4], suggesting that the mutagenesis was very effective.

The mutant library was screened for genes involved in tumor biology (*p53*, and *Blm*, encoding Bloom helicase), neurodegeneration (*Parkin*, encoding ubiquitin ligase), aging (*Sirt1*, encoding deacetylase), and miRNA metabolism (*Dcr-1*, encoding Dicer). Although a variety of mutation discovery technologies have been established for targeted retrieval of induced mutations [11-14], we chose to use dideoxy resequencing of PCR-amplified target sequences for routine mutation discovery [15], as this technology is robust and can be automated very well at both the experimental and data interpretation levels [16]. Most importantly, it provides highly informative data about the exact location and nature of the mutation.

We screened the complete library for 10 different amplicons covering 20 exons in 5 different genes (Table 2). In total, about 22 Mbp were screened and 64 independent mutations were identified (Table 3). The average ENU-induced mutation frequency for the library was found to be 1 mutation per 345,000 bp, similar to what was found for reverse genetic screens in zebrafish [12]. We retrieved highly likely loss-of-function mutations for four out of five genes screened by the identification of four nonsense and two splice site mutations. Although a full loss-of-function has to be demonstrated for each mutant individually, we refer to these mutants as knockouts in this paper. Furthermore, 38 missense mutations were found in the different genes (Tables 2 and 3), some of which could potentially result in a partial or complete loss-of-function or gain-of-function phenotype.

All nonsense and splice site mutants were recovered from the frozen sperm archive by *in vitro *fertilization (Table 4). A very high fertilization rate of more than 90% was consistently obtained following standard *in vitro *fertilization procedures, with only 7% to 33% of the fertilized eggs failing to develop and hatch. Genotyping tail fin tissue from a portion of F2 offspring revealed that the ratio of wild-type fish to mutant heterozygotes was about one-to-one, as expected (data not shown).

### *p53*^*E241X *^mutant characterization

**Figure 2:**
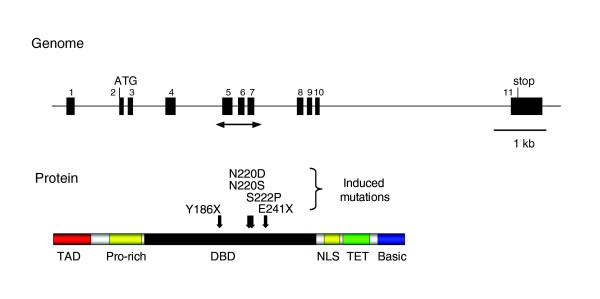
Target-selected mutagenesis of *Oryzias latipes p53 *gene. Genomic organization and protein structure of the medaka *p53 *gene. The region analyzed by PCR and dideoxy resequencing is indicated by bidirectional arrows. The ENU mutations are shown by solid arrows. Basic, basic regulatory region; DBD, DNA-binding domain; NLS, nuclear localization signal; Pro-rich, proline-rich domain; TAD, transactivation domain; TET, tetramerization domain.

We identified seven induced mutations in the medaka *p53 *gene [17], including three missense mutations, one splice site, and two nonsense mutations (Figure 2). The *p53*^*E241X *^allele is a G to T substitution that results in the alteration of Glu241 to a stop codon, whereas the *p53*^*Y186X *^allele is a T to A substitution that alters Tyr186 to a stop codon. Both were presumed to result in a truncated protein that terminates prematurely in the midst of a DNA-binding domain. These proteins retain the amino-terminal transactivation domain but lack the nuclear localization signal and tetramerization domain required for full activity. Furthermore, no alternative splicing variants involving these mutation-containing exons are known in any species, indicating that these nonsense mutations are most likely to result in a null phenotype. All three missense mutations are at highly conserved residues within the DNA-binding region, but more detailed characterization will be needed to conclude anything about their effect on protein function.

**Figure 3:**
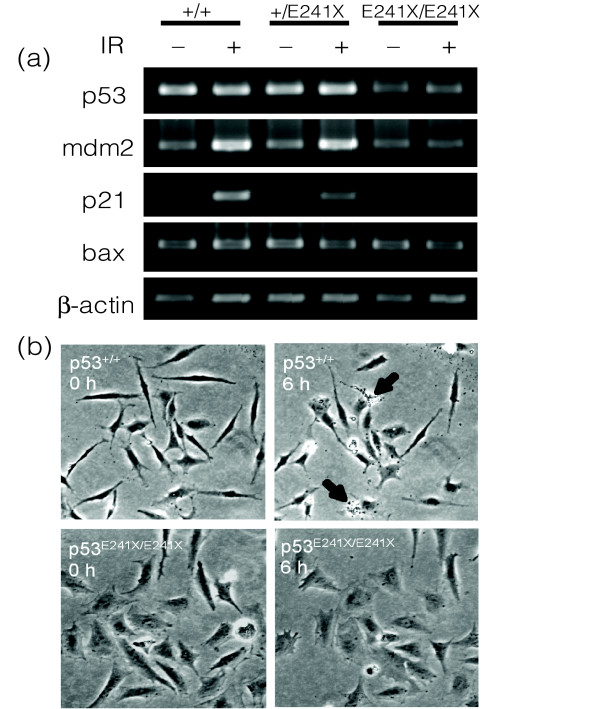
Radiation-induced *p53 *target gene induction and apoptosis. **(a) **Impaired IR-induced transactivation of target genes. Using semi-quantitative RT-PCR, induction of *Mdm2 *and *p21 *upon γ-irradiation can readily be observed in wild-type and heterozygous embryos, but is absent in animals homozygous for the *p53 *mutant allele. **(b) **Suppression of apoptosis in primary cultured cells. Primary cells derived from *p53*^*E241X/E241X *^and *p53*^+/+ ^embryos were irradiated with 10 Gy of ionizing radiation and observed by time-lapse microscopy. The apoptotic cells from homozygous embryos with fragmented nuclei are indicated with arrows.

Impaired target gene induction upon DNA damage is one of the phenotypes that is expected in a *p53 *knockout animal [18]. *p53*^*E241X/E241X *^embryos were γ-irradiated and the induction of *p21*, *Mdm2 *and *Bax *genes was examined by RT-PCR. As expected, no increase of these target genes was observed in *p53*^*E241X/E241X *^homozygous fish, while control fish clearly showed upregulation of *p21 *and *Mdm2 *transcription level in response to ionizing radiation (IR), (Figure 3a). Interestingly, the basal level of the *p53 *transcript was decreased in *p53*^*E241X/E241X *^fish. This could be due to nonsense-mediated decay [19] of mutant RNA, a phenomenon that is frequently observed in ENU-induced nonsense mutants (E Cuppen, unpublished observations), although an autoregulatory mechanism cannot be excluded. The same results were obtained for the second nonsense allele (*p53*^*Y186X/Y186X*^; data not shown). Next, we investigated whether IR-induced apoptosis was affected in *p53*^*E241X/E241X *^mutants. Primary cell cultures were derived from wild-type and *p53*^*E241X/E241X *^fish, γ-irradiated, and observed by time-lapse video microscopy for apoptosis. While 13.2% (15 out of 142 cells counted) of *p53*^+/+ ^cells underwent apoptosis, none of the *p53*^*E241X/E241X *^cells (0 out of 121 cells) showed fragmentation of the nucleus (Figure 3b). These results are consistent with a complete loss-of-function phenotype of p53 in these medaka mutants.

**Figure 4:**
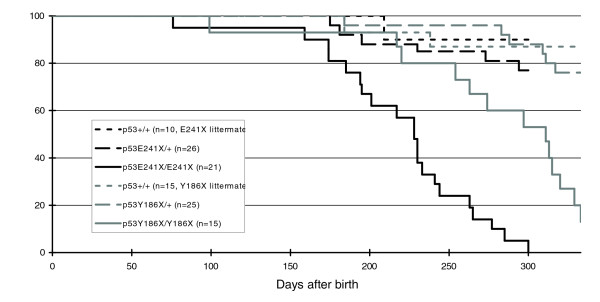
Survival curve of *p53 *mutant medaka. The viability of wild-type (dotted lines), heterozygote (dashed lines), and homozygote (solid lines) littermates of the *p53*^*E241X *^(black) and *p53*^*Y186X/Y186X *^(grey) fish was monitored for 10 months.

To monitor for spontaneous tumorigenesis, *p53 *knockout (*p53*^*E241X/E241X*^, *n *= 21), heterozygote (*p53*^+/*E241X*^, *n *= 26), and wild-type (*p53*^+/+^, *n *= 10) littermates were raised to adulthood to monitor for spontaneous tumorigenesis. Only a single *p53*^+/+ ^fish died within 10 months after birth with no obvious signs of cancer (Figure 4). Heterozygous fish developed some tumors during the course of observation (two out of the five fish that died during the first ten months had clear tumors), but the mortality rate was relatively low. In contrast, a dramatic tumor predisposition was observed in the homozygotes, with the first incidence of tumorigenesis observed already at 2.5 months of age. The frequency of tumor formation increased after 5 months of age, resulting in a median lifespan of 228 days. All homozygous fish died within 10 months and 11 out of the 21 animals had clear tumors. The real tumor rate is most likely higher, as a significant part of the dead fish could unfortunately not be examined properly, due to rapid decomposition. It should be mentioned that at least 2 out of the 21 *p53*^*E241X/E241X *^fish died without any macroscopic signs of tumors. The *p53*^*Y186X/Y186X *^fish developed tumors as well but at a lower rate compared to the *p53*^*E241X/E241X *^mutant. The median lifespan was also slightly increased (311 days), but was still much shorter than for wild-type fish (Figure 4). The difference in tumorigenesis between the two different nonsense alleles is not clear at this moment. We cannot exclude the possibility that co-segregating ENU mutations affect the predisposition to develop tumors in the *p53*^*E241X *^background. The analysis of heteroallelic *p53*^*E241X/Y186X *^fish and/or analysis of further outcrossed lines should resolve this issue.

**Figure 5:**
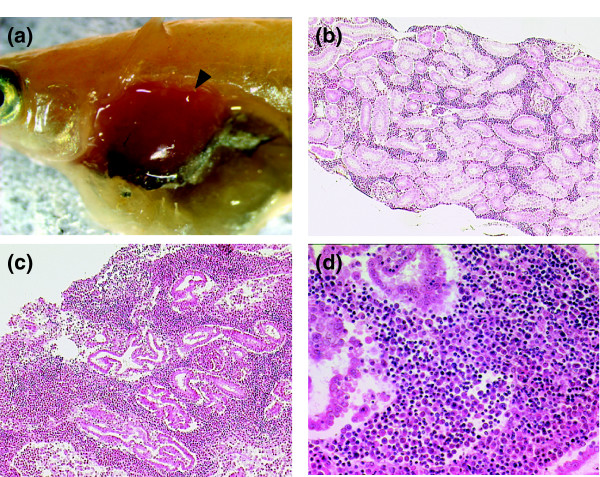
Typical kidney tumor as found in *p53*^*E241X/E241X *^homozygous fish. **(a) **A stereoscopic view of the kidney tumor identified in a 2.5 month old homozygous *p53*^*E241X/E241X *^fish. **(b-d) **Hematoxylin-eosin staining of normal (b) and neoplastic (c) kidney of medaka. Note that the interstitial tissue is infiltrated with numerous hematopoietic cells destroying the normal architecture of renal tubules. The higher magnification shows the mixture of small lymphocytes with little cytoplasm and the plasmacyte-like cells with large basophilic cytoplasm (d).

**Figure 6:**
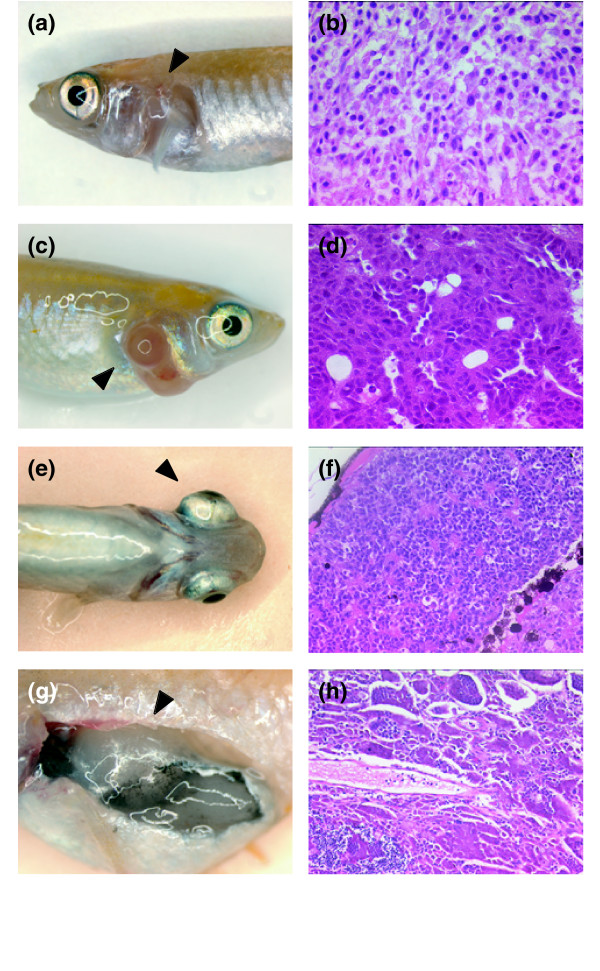
Various tumors that developed spontaneously in *p53 *medaka knockouts. **(a,b) **The tumor that arose in the left gill of a *p53*^*E241X*/+ ^fish with the lymphomatous infiltrate, consistent with the diagnosis of thymic lymphoma. **(c,d) **Adenocarcinoma found in the right gill of a *p53*^*E241X/E241X *^homozygous fish. **(e,f) **Retinoblastoma in the right eye of a *p53*^*E241X/E241X *^homozygous fish. Note the rosette-like structures throughout the tumor. **(g,h) **A germ cell tumor found in the anterior upper part of the peritoneal cavity of a *p53*^*E241X/E241X *^homozygous fish. All fish presented here died or were sacrificed at around 8 months of age. Arrowheads indicate tumors. Hematoxylin-eosin staining, original magnification: (b,d) 100×; (f,h) 10×.

Stereoscopic as well as histological characterization of tumor-bearing *p53*^*E241X *^mutant fish revealed a wide variety of tumor types in kidney, eye, brain, intestine, gill, thymus and testis (Figures 5 and 6). In one case, where kidney is the primary origin, lymphoid cells spread throughout the interstitial space, destroying the normal architecture of renal tubules and glomeruli (Figure 5). This is consistent with the observation that the teleost kidney is developmentally a mesonephros, which is the site for hematopoiesis in adult fish and is thought to function analogously to the bone marrow in mammals [20]. Considering a very low natural occurrence of tumors in young medaka (<0.01%) and the propensity of medaka to liver tumors [21], the diversity in tumor types and the high incidence of tumors observed in *p53*-deficient fish implicate that the *p53 *knockout medaka are highly susceptible to spontaneous tumorigenesis compared to their *p53*-proficient littermates, even though the number of fish examined in this study was relatively small.

In *p53*-deficient zebrafish, peripheral nerve sheath tumors were found to predominate [22]. The difference in tumor spectrum may be caused by the type of mutation introduced in the genome, namely a missense mutation at a conserved residue in zebrafish versus a nonsense mutation in medaka, or by the presence of organism-specific secondary genes that are differentially involved in tumor susceptibility. This tissue specific tumor development in different species is of great interest as this phenomenon is also found in mammals: in Li-Fraumeni syndrome patients, caused by mutations in the human *p53 *gene, breast cancer and sarcomas are most common, whereas *p53 *knockout mice develop T cell lymphomas [23,24]. Such differences strengthen the need for parallel studies in multiple model organisms.

We identified a nonsense mutation that results in a truncated Parkin protein at Tyr314, eliminating the inbetween RING domain (IBR) and the second RING domain (RING2), which are critical for its ubiquitin ligase activity [25]. Interestingly, a similar mutation, which results in Parkin protein truncation at Glu311, has been found in a human juvenile parkinsonism patient [26]. For the *Blm *gene, the premature stop codon was introduced at position Glu497, which removes the entire critical helicase domain. Again, a similar 515 amino acid-long truncated protein has been reported in a human disease case that results from a 1 bp insertion prior to the helicase domain [27]. It should be noted that the complete knockout of the *Blm *gene results in embryonic lethality in mice [28], while *Blm *mutant medaka fish are viable, similar to human. We expect that the medaka mutants of the Parkinsonism and Bloom syndrome genes may serve as valuable disease models, and are currently characterizing their phenotypes in detail.

## Conclusion

The estimated evolutionary distance of 110 to 200 million years between medaka and zebrafish, and the partial or whole genome duplication that occurred in the common ancestor of teleosts with subsequent diversification events in the different lineages make medaka a suitable animal for comparative approaches [1,29]. The establishment of knockout technology for medaka, as described here, adds significantly to the experimental possibilities in this emerging model organism. A compact genome that lacks the complex repetitive elements observed in zebrafish, and the availability of several inbred strains [30] make the medaka fish model especially suited for genome-based analyses. Furthermore, in contrast to zebrafish, which inhabit tropical areas, medaka passes the winter in Japan, surviving water temperatures as low as 4°C [1]. This opens the possibility for heat- or cold shock-based experiments. Considering this, the missense mutations retrieved by our target-selected mutagenesis approach could be very interesting as some of them may represent temperature sensitive alleles. Among the mutants we recovered, N220S and N220D of *p53 *are of particular interest, because Asn220 is located next to the Zn-binding cysteine in loop 3, which is important for stabilization of p53 folding [31]. In fact, the change in the thermostability of human p53 protein has been observed for the mutation in Asn239 (equivalent to Asn220 of medaka p53) [32,33]. It would be interesting to examine the thermodynamics and temperature sensitive effect on the animal carrying these mutations.

Fish, like medaka and zebrafish, are becoming increasingly important models in biomedical research [1,29]. In relation to tumor biology, transgenic approaches have been shown to be valuable to induce cancers and leukemia in both zebrafish and medaka [34,35]. The *p53*-deficient medaka reported here and two other recently described target-selected knockouts in zebrafish [22,36] are unique in that the disease is caused by the loss of a tumor suppressor rather than overexpression or activation of an oncogene. The role of *p53 *in fish cancer has been questioned because mutations in the *p53 *gene have only rarely been found in naturally occurring or induced tumors in teleosts [37], but our results and the work by Berghmans *et al*. [22] clearly show that *p53 *also plays a general role in tumorigenesis in fish as 'a guardian of the genome'. Since it is known that tumor formation with oncogene or chemical mutagens is accelerated by *p53 *mutations [38], *p53*-deficient medaka fish are likely to become an important tool to understand the mechanisms underlying oncogenesis in general.

Taken together, the high ENU-induced mutation frequency and efficient mutation discovery, combined with the compact medaka genome and efficient cryopreservation and rederivation protocols, have resulted in the development of a highly effective approach for the routine generation of knockouts in medaka. More detailed phenotypic characterization of the retrieved mutants will undoubtedly provide valuable insight into the molecular mechanisms in which these genes are involved, and add to the versatility of the medaka animal model in general. Finally, the cryopreserved mutant library described here is expected to contain knockouts for most medaka genes, providing a valuable resource for the research community.

## Materials and methods

### Mutagenesis

*Kyoto-Cab*, a substrain of Cab, was mutagenized as described previously with slight modifications [4]. Males (102; G0) were treated weekly with 3 mM ENU (Sigma-Aldrich, St. Louis, MO USA) in 10 mM sodium phosphate buffer (pH 6.3) at 26°C for 1 h. After the third treatment with ENU, the G0 were crossed with wild-type females to monitor the recovery of fecundity. A month after the last ENU treatment, crosses with wild-type females were set up and fertilized eggs were left to develop to full term, resulting in the mutant F1 library (only males were kept). The number of offspring produced from a single mutagenized male founder varied from 1 to 239, presumably reflecting variability in ENU-induced damage to the testis. Ten mutagenized male founders were crossed with albino fish (*Heino*) to monitor the mutagenesis efficiency using a single locus test.

### Cryopreservation of sperm

The sperm from each F1 medaka was cryopreserved as described in Section 3.3.1 of the medaka protocols book [39]. The sperm was suspended in 60 μl of freezing medium (10% dimethylformamide in fetal calf serum) and was divided into 6 glass capillaries. The amount of sperm held in each capillary was enough to fertilize more than 100 eggs.

### Genomic DNA extraction

After removing the testis, fish were cut in two halves and kept at -80°C until DNA was extracted. The tail side of the fish was incubated overnight at 55°C in 500 μl of lysis buffer containing 10 mM Tris-HCl (pH 7.5), 1 mM EDTA (pH 8.0), 120 mM sodium chloride, 12 mM sodium citrate (pH7.0), 1% SDS, and 200 μg/ml of proteinase K (Sigma-Aldrich). The lysate was phenol extracted and precipitated with isopropanol. The DNA pellet was dissolved in 1 ml of TE (10 mM Tris pH 7.5, 1 mM EDTA, pH8.0). The concentration was adjusted to 40 ng/μl, aliquoted into 96 deep well plates, and stored at -20°C.

### PCR assay design

For *p53*, both the cDNA and genomic sequence available (NCBI accession AF212997) have been published [17,37]. For *Blm*, *Sirt1 *and *Parkin *the medaka cDNA sequences were determined using a combination of RT-PCR and rapid amplification of cDNA ends (RACE). Overall, the similarity of the encoded medaka proteins to their human counterparts was 80%, 80% and 90%, respectively, and the identity was 42%, 50% and 56%, respectively. The cDNA sequences were used to retrieve the genomic sequence from the draft medaka genome assembly [8]. For *Dcr-1*, the zebrafish protein sequence was used in combination with gene prediction algorithms to retrieve the genomic sequences required for amplicon design. For all genes, only a single ortholog of the human gene was identified in the medaka draft genome. All genes were imported in the laboratory information management system for the identification of mutations by sequencing or TILLING: LIMSTILL [40] to facilitate amplicon design for screening and mutation annotation. Oligonucleotides for the amplification of exons of interest by nested PCR were designed in LIMSTILL. Details on oligonucleotides used can be obtained from the authors upon request. For universal processing at the sequencing stage, the primers for the second PCR reaction contained M13 tails. The sequence of the universal tails and M13 oligonucleotides used for sequencing were: M13-Forward, TGTAAAACGACGGCCAGT; and M13-Reverse, AGGAAACAGCTATGACCAT.

### Discovery of induced point mutations by dideoxy resequencing of PCR amplicons

Genomic DNA stocks were diluted 25-fold and gridded out as 5 μl aliquots into 384 well plates. The first PCR (PCR1) was carried out using a touchdown thermocycling program (92°C for 60 s; 12 cycles of 92°C for 20 s, 65°C for 20 s with a decrement of 0.6°C per cycle, 72°C for 30 s; followed by 20 cycles of 92°C for 20 s, 58°C for 20 s and 72°C for 30 s; 72°C for 180 s; GeneAmp9700, Applied Biosystems, Foster City, CA, USA). This reaction contained 5 μl genomic DNA, 0.2 μM forward primer and 0.2 μM reverse primer, 400 μM of each dNTP, 25 mM tricine, 7.0% (w/v) glycerol, 1.6% (w/v) DMSO, 2 mM MgCl_2_, 85 mM ammonium acetate pH 8.7 and 0.2 U Taq polymerase in a total volume of 10 μl. After thermocycling, the PCR1 reactions were diluted with 25 μl water, mixed by pipetting, and 1 μl was used as template for the second round of PCR. The second PCR (PCR2) was done using a standard thermocycling program (92°C for 60 s; 30 cycles of 92°C for 20 s, 58°C for 20 s and 72°C for 30 s; 72°C for 180 s; GeneAmp9700, Applied Biosystems). PCR2 mixes contained 1 μl diluted PCR1 template, 0.1 μM forward primer, 0.1 μM reverse primer, 100 μM of each dNTP, 25 mM tricine, 7.0% (w/v) glycerol, 1.6% (w/v) DMSO, 2 mM MgCl_2_, 85 mM ammonium acetate pH 8.7 and 0.1 U Taq polymerase in a total volume of 5 μl. Several samples of each amplicon were tested on a 1% agarose gel containing ethidium bromide for the presence of the proper PCR fragment.

PCR2 products were diluted with 20 μl water and 1 μl was directly used as template for the sequencing reactions. Sequencing reactions, containing 0.12 μl BigDYE (v3.1; Applied Biosystems), 1.88 μl 2.5× dilution buffer (Applied Biosystems) and 0.4 μM universal M13 primer in a total volume of 5 μl, were performed using cycling conditions recommended by the manufacturer (40 cycles of 92°C for 10 s, 50°C for 5 s and 60°C for 120 s). Sequencing products were purified by ethanol precipitation in the presence of 40 mM sodium acetate and analyzed on a 96-capillary 3730XL DNA analyzer (Applied Biosystems), using the standard RapidSeq protocol on a 36 cm array. Sequences were analyzed for the presence of heterozygous mutations using PolyPhred [41] and manual inspection of the mutated positions. Every candidate mutation was verified by an independent PCR and resequencing read. Nucleotide variations that were present in more than two F1 fish were considered to be single nucleotide polymorphisms (SNPs) and, therefore, excluded from further analysis, while mutations found in only two animals were included, as examination of the breeding records revealed that, in most cases, these originated from the same mutagenized parent and are thus most likely to be derived from the same spermatogonial stem cell. These mutations are counted as a single mutation.

### *In vitro *fertilization

About 100 unfertilized eggs were squeezed out from the wild-type cab females. A single glass capillary containing 10 μl of sperm from the F1 fish identified in the screening was removed from the liquid nitrogen and thawed by placing at ambient temperature. Immediately after the sperm was thawed, the content was pushed out in balanced salt solution (BSS; 0.65% sodium chloride, 0.04% potassium chloride, 0.02% magnesium sulfate heptahydrate, 0.02% calcium chloride dihydrate, 0.00005% phenol red, 0.01% sodium hydrogen carbonate, pH 7.3) and incubated with the eggs for 20 minutes with occasional pipetting. The eggs that were not fertilized were removed 3 h later, and BSS was replaced with 0.03% Red Sea salt water. The eggs were incubated at 28°C until they hatched. The quality of thawed sperm and the fertilization rate was checked under the microscope. Only a single cryopreserved straw (out of six straws frozen in total) was needed for successful recovery of each mutation of interest. For each *in vitro *fertilization, between 66 and 88 fish were obtained that developed to adulthood. Progeny were genotyped by sequencing and heterozygous fish carrying the mutation of interest were incrossed to obtain homozygous fish.

### *p53 *target gene induction

For most of the studies, the *p53*^*E241X *^allele was used. F2 *p53*^+/*E241X *^heterozygous fish resulting from the *in vitro *fertilization were incrossed to produce F3 progeny of all genotypes. Four days post-fertilization, F3 embryos were irradiated with 20 Gy of ionizing radiation using ^137^Cs (0.02 Gy/s, Gammacell 40, Atomic Energy of Canada Limited Industrial Products, Ontario). Six hours later, the embryos were frozen in liquid nitrogen and RNA was extracted by Trizol (Invitrogen, Carlsbad, CA, USA) according to the manufacturer's instruction. The embryos were genotyped by PCR and resequencing of the simultaneously extracted genomic DNA. cDNA was synthesized from each genotype using SuperScript III (Invitrogen). The mRNA expression levels were determined by PCR reactions (94°C for 1 minute; predetermined cycles of 94°C for 30 s, 55°C for 20 s, 72°C for 30 s). The numbers of cycles used were: *β-actin*, 15; *Mdm2*, 24; and *p53*, *p21 *and *Bax*, 26. Details on oligonucleotides used can be obtained from the authors upon request.

### Apoptosis assay

Primary cell cultures derived from *p53*^*E241X/E241X *^and *p53*^+/+ ^embryos were obtained as described previously [42]. Cells (1.5 × 10^5^) were inoculated in a 35 mm dish and irradiated with 10 Gy of γ-rays. The cells were monitored for fragmentation using a IX81 inverted microscope (Olympus, Tokyo, Japan) controlled by IPLab software (BD Biosciences, Rockville, MD, USA) from zero to eight hours after γ-irradiation.

### Histology

After tumors were observed under the stereomicroscope, fish were fixed in 4% paraformaldehyde for 24 hours and embedded in paraffin. Tissue sections were stained by standard hematoxylin-eosin staining. Photographs of the slides were obtained by a VC4500G digital camera (Omron, Kyoto, Japan) mounted on an ECLIPSE E800 microscope (Nikon, Tokyo, Japan).

## Figures and Tables

**Table 1:** Statistics on the mutant medaka library generation

	Library generation	Specific locus test
G0	87	9*
Fertilized eggs^†^	26,226	1,360
F1	5,771 mature males	5 albino mutants

*The fish used for specific locus test were eventually mated to wild-type females and overlap with 87 fish that were used for library generation. ^† ^The number of fertilized eggs includes those that died during embryogenesis.

**Table 2:** Medaka mutant library* screening statistics

Gene	Exons	Exons screened	Amplicons^†^	Base-pairs screened^‡^	Exonic			Intronic		Total	Mutation rate
							
					Stop	Missense	Silent	Intron	Splice		
*Blm*	23	2	2	3,129,006	1	4	0	1	0	6	1/521,501
*p53*	11	3	1	1,854,603	2	3	0	1	1	7	1/264,943
*Sirt1*	9	5	2	5,767,496	0	12	0	2	1	15	1/384,500
*Dcr-1*	27	7	4	7,879,290	0	16	4	7	0	27	1/291,826
*Parkin*	11	3	1	3,461,661	1	3	3	2	0	9	1/384,629
Total	81	20	10	22,092,056	4	38	7	13	2	64	1/345,188

*The mutant library consists of 5,771 cryopreserved male progeny from ENU-mutagenized fish. ^†^Due to the compact medaka genome architecture, multiple exons can often be amplified and sequenced from a single amplicon. ^‡^Determined by counting all bases in the resequencing reads that were read with phred quality >20.

**Table 3:** Detailed overview of the induced mutations retrieved from the mutant medaka library

Number	Exon	Sequence context	Amino acid change	Type of mutation
**Dicer (*Dcr-1*)**				
1	10_11	5'-GATCCTTAGG (A>G) ACAAATGCTC-3'	N578D	Substitution
2	10_11	5'-GTGGTTGACG (A>G) TGACAACATC-3'	D597G	Substitution
3	10_11	5'-ACCGTCAACA (C>A) AGCCATCGGT-3'	T619K	Substitution
4	10_11	5'-CGTCAACACA (G>A) CCATCGGTCA-3'	A620T	Silent
5	10_11	5'-CAGGTACCTG (C>T) CCTGCTTTGA-3'		Intron
6	10_11	5'-CCTGCCCTGC (T>A) TTGATGTGGA-3'		Intron
7	10_11	5'-AGAATTAACT (T>A) CAACTCAACA-3'		Intron
8	16_17	5'-ATTTTTGACT (T>A) GAATAGTTGG-3'		Intron
9	16_17	5'-GAGGCTCGCA (C>T) TGGCATTCCT-3'	T897I	Substitution
10	16_17	5'-CGCACTGGCA (T>G) TCCTACCACT-3'	I899S	Substitution
11	16_17	5'-ACTACCAGGA (C>A) GCTGTCATCA-3'	D919E	Substitution
12	16_17	5'-GCTCCTTCAG (T>A) GAAACTCTTG-3'		Intron
13	16_17	5'-TCTCCATAGA (T>A) ATCGTAACTT-3'	Y926N	Substitution
14	16_17	5'-CCATAGATAT (C>T) GTAACTTTGA-3'	R927C	Substitution
15	16_17	5'-GCCACTCAGC (A>G) AGTTTCCTTC-3'	K949E	Substitution
16	16_17	5'-TTCCTTCACC (A>T) GAATACGAGA-3'	P953P	Silent
17	16_17	5'-ACCTGTCAAA (T>A) CTGAACCAGC-3'	N972K	Substitution
18	20a	5'-GGTTTTTGTG (T>C) CAGATATCCA-3'		Intron
19	20a	5'-CCATTGACAA (C>A) AAAGCTTACA-3'	N1094K	Substitution
20	20a	5'-AAGCTTACAG (T>A) TCTTGCTCCG-3'	S1098R	Substitution
21	20a	5'-TTGCTCCGAG (T>C) CCTGCAGCGA-3'	S1103P	Substitution
22	20a	5'-GCTCAGAACC (T>G) GCCCTCTCAG-3'	P1120P	Silent
23	20a	5'-CCTTCACCAA (C>T) CTGACAGCTG-3'	P1168S	Substitution
24	22b	5'-AATAAGGCCT (A>G) CCTGCTGCAA-3'	Y1635C	Substitution
25	25_26	5'-AGGAAGAGGA (C>T) ATTGAGGTCC-3'	D1754D	Silent
26	25_26	5'-TTCATCACTG (T>A) TGTTGGAGAT-3'		Intron
27	25_26	5'-CTGCTGGAGA (T>A) GGAGCCGGAA-3'	M1813K	Substitution
				
**p53**				
1	5_6_7	5'-TCCCTTTTCT (C>T) CATCGACTGT-3'		Intron
2	5_6_7	5'-TGGCCCAGTA (T>A) TTTGAAGACC-3'	Y186X	Truncation
3	5_6_7	5'-CTACATGTGT (A>G) ACAGCTCGTG-3'	N220D	Substitution
4	5_6_7	5'-TACATGTGTA (A>G) CAGCTCGTGC-3'	N220S	Substitution
5	5_6_7	5'-GTGTAACAGC (T>C) CGTGCATGGG-3'	S222P	Substitution
6	5_6_7	5'-TCTGGAAACC (G>T) AGTAAGTTTA-3'	E241X	Truncation
7	5_6_7	5'-GGAAACCGAG(T>C)AAGTTTAGTC-3'		Splice
				
**Sirt1**				
1	2_3_4	5'-CGATGACGGA (T>A) CCTCTCATGC-3'	S138T	Substitution
2	2_3_4	5'-CTAGTTCCAG (C>G) GACTGGACTC-3'	S144R	Substitution
3	2_3_4	5'-AGTTCCAGCG (A>G) CTGGACTCCG-3'	D145G	Substitution
4	2_3_4	5'-AGCGACTGGA (C>T) TCCGCAGCCC-3'	T147I	Substitution
5	2_3_4	5'-CAGCCCCAGA (T>A) CGGTCAGAAT-3'	I152N	Substitution
6	2_3_4	5'-AAGCCGTTGT (G>T) AGCTCAGGTG-3'		Intron
7	2_3_4	5'-CCCGAGACCA (T>C) ACTCCCACCC-3'	I179T	Substitution
8	2_3_4	5'-CTGTGGCAGA (T>C) CATCATCAAC-3'	I192T	Substitution
9	2_3_4	5'-ATCATGGTTC (T>C) GACCGGTGCA-3'	L227P	Substitution
10	2_3_4	5'-CGGTGCAGGT (G>T) TAGGTGTTTC-3'		Splice
11	2_3_4	5'-TAAAGAAACG (G>A) TAAACACCGG-3'		Intron
12	2_3_4	5'-CGGCTTGCTG (T>C) CGACTTTCCC-3'	V253A	Substitution
13	5_6	5'-AACATCGACA (C>A) GCTGGAACAA-3'	T317K	Substitution
14	5_6	5'-TGCGACGGCT (T>C) CCTGTCTCGT-3'	S338P	Substitution
15	5_6	5'-CGTTTGTAAA (C>A) ACAAAGTGGA-3'	H344N	Substitution
				
**Blm**				
1	5_6	5'-AGCAGTAGGG (C>T) AATCTGTGTG-3'	A477V	Substitution
2	5_6	5'-TGTGACTCTC (T>G) ATCAACTCCC-3'	L489R	Substitution
3	5_6	5'-ACTTCTAAAA (C>T) AACCTTGTTT-3'	Q497X	Truncation
4	5_6	5'-TTTCTCAGAG (A>G) GCACAAGTCG-3'	S503G	Substitution
5	7	5'-TATTTTCTAT (C>T) TTCATTCAGA-3'		Intron
6	7	5'-CTTGATGCCC (A>G) CAGGTTGGTG-3'	T670A	Substitution
				
**Parkin (*Park2*)**				
1	9_10_11	5'-ATGCACGGTA (C>G) CAGCAATATG-3'	Y314X	Truncation
2	9_10_11	5'-GACTCATGTG (T>C) CCGGCACCTG-3'	C331C	Silent
3	9_10_11	5'-AGGGTGGAGT (G>T) TGAGAGACAG-3'	C351F	Substitution
4	9_10_11	5'-GCTGTGGCTT (T>A) GTCTTCTGTA-3'	F359L	Substitution
5	9_10_11	5'-TTTTGTGATG (A>T) CATTGCCGTG-3'		Intron
6	9_10_11	5'-GTCTTATTCA (G>A) GAGATGACCA-3'	Q410Q	Silent
7	9_10_11	5'-TCTCCACCTG (C>T) AGGTGGCTGC-3'		Intron
8	9_10_11	5'-TGCACATGCA (T>C) TGTGCTCTGT-3'	H433H	Silent
9	9_10_11	5'-AGGGAGTGCA (T>A) GGGAAACCAC-3'	M454K	Substitution

**Table 4:** *In vitro *fertilization statistics

Mutants	Eggs used	Fertilized*	Hatched*
*p53*^*Y186X*^	101	100 (99)	88 (87)
*p53*^*E241X*^	106	105 (99)	81 (76)
*p53*^*splice*^	101	99 (98)	86 (85)
*Sirt1*^*splice*^	99	93 (94)	84 (85)
*Blm*^*Q497X*^	103	98 (95)	66 (64)
*Parkin*^*Y314X*^	98	88 (90)	82 (84)

*The number in parentheses indicates the percentage of fertilized/hatched embryos.
